# Transient attention equally reduces visual crowding in radial and tangential axes

**DOI:** 10.1167/jov.22.9.3

**Published:** 2022-08-03

**Authors:** Bahiyya Kewan-Khalayly, Marta Migó, Amit Yashar

**Affiliations:** 1 Department of Special Education, University of Haifa, Haifa, Israel; 2 Department of Psychiatry, Massachusetts General Hospital, Harvard Medical School, Boston, MA, USA; 3 Department of Special Education, University of Haifa, Haifa, Israel; 4 The Edmond J. Safra Brain Research Center for the Study of Learning Disabilities, University of Haifa, Haifa, Israel

**Keywords:** crowding, radial–tangential anisotropy, attention, critical spacing, spatial vision

## Abstract

Crowding refers to the failure to identify a peripheral object due to its proximity to other objects (flankers). This phenomenon can lead to reading and object recognition impairments and is associated with macular degeneration, amblyopia, and dyslexia. Crucially, the maximal target–flanker spacing required for the crowding interference (critical spacing) increases with eccentricity. This spacing is also larger when target and flankers appear along the horizontal meridian (radial arrangement) than when the flankers appear above and below the target (tangential arrangement). This phenomenon is known as radial–tangential anisotropy. Previous studies have demonstrated that transient attention can reduce crowding interference; however, it is still unclear whether and how attention interacts with radial–tangential anisotropy. To address this issue, we manipulated transient attention by using a cue at either the target (valid) or the fixation (neutral) location, in both radial and tangential target–flanker arrangements. Results showed that critical spacing was larger in the radial than in the tangential arrangement and that cueing the target location improved performance and reduced the critical spacing for both radial and tangential arrangements to the same extent. Together, our findings suggest that transient spatial attention plays an essential role in crowding but not in radial–tangential anisotropy.

## Introduction

Visual crowding describes the phenomenon where an object becomes more difficult to identify when it is surrounded by other objects (flankers) rather than when it is by itself (Pelli, 2008; Whitney & Levi, 2011). Although mostly unnoticeable, crowding can happen at any location in the visual field, including the fovea (Clark, Intoy, Yang, & Poletti, 2020), but it is more predominant in peripheral vision (Levi, 2008). Other phenomena hindering flankered object perception include masking, lateral interaction, and surround suppression. However, crowding is an important issue associated with slow and faulty reading (Whitney & Levi, 2011) and is common among clinical populations with macular degeneration, amblyopia, and dyslexia, making it particularly urgent to study (Gori & Facoetti, 2015).

The spatial extent of crowding is often measured by the minimum spacing between target and flankers required for target recognition without interference. Researchers often refer to this spacing as the critical spacing of crowding. The critical spacing scales with eccentricity. That is, as target eccentricity increases, the critical spacing becomes larger (Bouma, 1970; Pelli, Palomares, & Majaj, 2004). Prior research has set this critical spacing at around 30% to 70% of the stimuli eccentricity (Bouma, 1970; Pelli et al., 2004; Strasburger, Rentschler, & Juttner, 2011; Whitney & Levi, 2011).

Recent studies have shown that crowding can occur at various levels of visual processing (e.g., Jimenez, Kimchi, & Yashar, 2022; Manassi & Whitney, 2018). For example, in addition to basic features, crowding can occur during the processing of complex stimuli, such as abstract shapes (Kimchi & Pirkner, 2015; Pirkner & Kimchi, 2017), everyday objects (Wallace & Tjan, 2011), faces (Farzin, Rivera, & Whitney, 2009; Louie, Bressler, & Whitney, 2007; Martelli, Majaj, & Pelli, 2005), or words (Martelli et al., 2005). Moreover, stimulus grouping and stimulus configuration can modulate crowding effects (e.g., Banks & White, 1984; Jimenez et al., 2022; Livne & Sagi, 2007; Malania, Herzog, & Westheimer, 2007). For example, recently, Jimenez et al. (2022) showed that grouping into a global configuration that forms an illusory shape interacted with the crowding of local features, suggesting that crowding co-occurs across multiple levels of visual processing. However, the underlying processes of crowding are still unknown.

Many crowding models rely on a pooling process, in which both target and flanker features are integrated together (Parkes, Lund, Angelucci, Solomon, & Morgan, 2001). Other models suggest a substitution process, in which target and flanker location information is lost, leading observers to confuse the target with a flanker (Freeman, Chakravarthi, & Pelli, 2012). However, another possible explanation for crowding involves attention. This account suggests that crowding happens due to limitations in the spatial resolution of attention, which is more limited in the visual periphery. Hence, observers become unable to selectively attend to the targets without also attending to the irrelevant flankers (Intriligator & Cavanagh, 2001; Tripathy & Cavanagh, 2002). Nevertheless, only a few studies have directly investigated the role of attention in crowding and its characteristics.

An important characteristic of crowding is its contingencies on the spatial layout of the flankers, meaning that crowding interference depends on the flanker and target arrangement (Strasburger, 2020). Two phenomena demonstrate this: (1) inner–outer asymmetry and (2) radial tangential anisotropy. Inner–outer (or “in–out”) asymmetry in the radial arrangement refers to the stronger interference created by the outer flanker than the inner one (Petrov, Popple, & McKee, 2007; Shechter & Yashar, 2021). Radial–tangential anisotropy refers to the phenomenon where crowding becomes 2 to 2.5 times more likely to happen when flankers are arranged radially (i.e., along the radius line drawn from the center of the visual field to the target) than tangentially (i.e., flankers are positioned above and below the target, perpendicular to the radius line) (Greenwood, Szinte, Sayim, & Cavanagh, 2017; Mareschal, Morgan, & Solomon, 2010; Petrov & Meleshkevich, 2011b; Toet & Levi, 1992). However, the underlying processes of radial–tangential anisotropy, particularly the role of spatial attention, are still unknown.

Previous explorations of spatial attention on crowding have yielded inconsistent results (Huckauf & Heller, 2002; Scolari, Kohnen, Barton, & Awh, 2007; Strasburger, 2005; Strasburger & Malania, 2013; Van der Lubbe & Keuss, 2001; Yeshurun & Rashal, 2010). On the one hand, some studies have failed to show an attentional effect on crowding errors beyond the overall effect of attention on performance (Scolari et al., 2007; Strasburger & Malania, 2013). For example, Strasburger and Malania (2013) found a pre-cue effect on target contrast but not on crowding misreport errors (reporting a flanker instead of a target). However, on the other hand, Yeshurun and Rashal (2010) demonstrated an attentional effect on crowding critical spacing by manipulating transient attention—fast covert (without eye movements) spatial attention (Carrasco, 2011). A key difference between studies that showed an attentional cueing effect and studies that did not was the location of the cue with respect to the target. For example, in a study that used a tangentially arranged target–flanker display, Scolari et al. (2007) failed to show critical spacing reduction by a peripheral cue that appeared at the location of the target. In contrast, Yeshurun and Rashel (2010), who also used a tangential target–flanker arrangement, demonstrated a critical spacing reduction of about 0.5° to 0.75° by a valid cue that appeared at an inner location than the target (i.e., a location between the center of the screen and a peripheral target). This finding suggests that transient attention increases the spatial resolution process that is involved in tangential crowding.

In radial crowding, Kewan and Yashar (2021) showed that the peripheral cue effect in crowding is contingent on the eccentricity of the cue with respect to the target location. Specifically, they showed that participants often misreported the outer flanker feature instead of the target feature (feature misreport errors). However, cueing the target location did not reduce feature misreport errors, and cueing the outer flanker location (a more eccentric location than the target) increased feature misreport errors. Importantly, cueing the inner flanker location in a radial arrangement (a less eccentric location than the target) decreased feature misreport errors. These findings are consistent with previous work demonstrating the role of attention in the inner–outer asymmetry and in radial crowding (Petrov & Meleshkevich, 2011a). However, no study, to our knowledge, has tested the effect of attention manipulation on the spatial extent of radial crowding. Moreover, although one study demonstrated reduction of the radial–tangential anisotropy by learning (Malania, Pawellek, Plank, & Greenlee, 2020), no study has investigated whether and how spatial attention reduces the radial–tangential anisotropy of crowding.

Here, we addressed these issues by investigating the effects of a peripheral pre-cue on the critical spacing of either radial or tangential crowding. Participants performed an orientation discrimination task of a T-shaped letter. To assess crowding spatial extent, we varied target–flanker spacing and flanker arrangement (either radial or tangential). Following previous investigations of spatial attention in crowding (e.g., Yeshurun & Rashal, 2010), we used two different cue conditions (valid vs. neutral) to manipulate covert transient spatial attention. First, we hypothesized that spatial attention would reduce the spatial extent of crowding—namely, we believed that the critical spacing would be smaller in valid compared with neutral trials. Furthermore, spatial attention did indeed play a role in radial–tangential anisotropy. We predicted that the attentional effect on the spatial extent would be contingent on the flanker arrangement axis. For example, attentional shifts on the radial axis could be more biased and inaccurate than on the tangential axis. In this case, pre-cueing attention may reduce this bias, leading to a larger reduction of critical spacing in the radial arrangement and, hence, smaller radial–tangential anisotropy.

## Methods

### Participants

Sixteen students (nine males; age range, 19–35 years, *M* = 27.75, *SD* = 4.81) from the University of Haifa participated in this study, either in exchange for course credit or payment of 50 shekels (around $14) per hour. Based on previous literature, we estimated that a sample size of 12 participants was required to detect a crowding effect with 95% power, given a 0.05 alpha (Yashar, Wu, Chen, & Carrasco, 2019). However, we collected data from four additional participants to account for possible dropouts or technical difficulties. All participants were blind to the research question and reported normal or corrected-to-normal vision and no attention deficits. Informed written consent was obtained from all participants before they began the study, and all practices and procedures were approved by the University Committee on Activities Involving Human Subjects at Haifa University (no. 226/20).

### Apparatus

Stimuli were presented using MATLAB software (MathWorks, Natick, MA) and Psychtoolbox and were displayed on a gamma-corrected 21-inch cathode-ray tube monitor (with 1280 × 960 resolution and 85-Hz refresh rate). EyeLink 1000 (SR Research, Kanata, Ontario, Canada), an infrared eye tracker, was used to monitor and record eye movement, and a SpectroCAL MKII spectroradiometer (Cambridge Research Systems, Cambridge, UK) was utilized to calibrate brightness and color. Participants were individually tested in a dimly lit room and prompted to use a keyboard to generate responses. Finally, a chinrest was used to ensure that all participant were 57 cm away from the computer monitor.

### Stimuli and procedure

**Figure 1.**
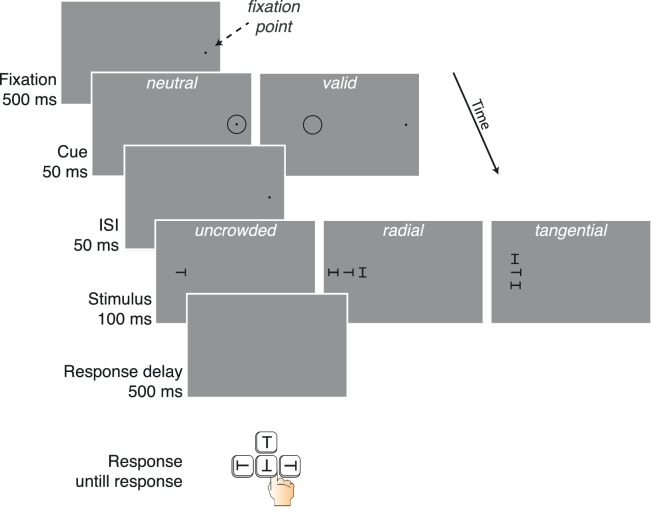
Illustration of the sequence of events within a trial. After a fixation point was displayed, a valid or neutral cue appeared briefly before the stimuli. The participant was asked to maintain eye fixation for the entire duration of the stimulus presentation and report the orientation of the target. An eye tracker was used to monitor eye fixation. In this experiment, the fixation point was presented at the center of the screen.

Figure 1 illustrates the paradigm of the experiment. All stimuli were colored black (luminance 0.0073 cd/m^2^) and presented on a gray background (53 cd/m^2^). First, participants were asked to fixate their gaze on the location of the fixation mark. This fixation mark was a centered black dot (subtending 0.24 degree of visual angle), which appeared on the screen for 500 ms and continued to appear until the participant maintained fixation for 300 ms. Following observer fixation, a cue appeared on the screen for 50 ms. The cue was a black ring (1-pixel pen width) subtending 1° of diameter. In the neutral cue condition, the cue circle appeared at the center of the screen. In the valid cued condition, the cue appeared 5.9° away from the center of the screen, on the horizontal meridian, in the same hemifield as the target. An interstimulus interval (ISI) of 50 ms followed the cue, and the target display then appeared for 100 ms. In the crowded display trials, three letter shapes (each subtended 0.75 degree of visual angle) appeared on the screen: one target and two flankers. Note that eye movement was tracked throughout the task, and all trials in which participants did not fixate on the center of the screen were removed from analysis.

The target was a “T” shape, oriented upright (0°), inverted (180°), or tilted to the left (270°) or the right (90°), and it was presented at an eccentricity of 7° on the horizontal meridian either to the right or to the left of the fixation mark. On valid trials, the cue was inner to the target and at 1.1° center-to-center distance from the target. This location was chosen based on previous work revealing that the most effective cue location in producing an attentional effect was an inner cue (Kewan & Yashar, 2021). Flankers were two “H” shapes, either upright or tilted 90°. On half of the crowded display trials, the flankers were positioned radially: one to the right and one to the left of the target. On the other half of the trials, the flankers were positioned tangentially: one above and one below the target. In each crowded display trial, both flankers were equally spaced from the target. Target–flanker center-to-center spacing was 1.1°, 2°, 3°,4°, 5°, 6°, 8,° or uncrowded (target alone). Target and flankers were always black. After 500 ms, the response period began, and the monitor displayed a blank screen.

Participants were instructed to report the orientation of the target by pressing on one of four designated keys on the keyboard (each key representing one of the four possible target orientations). Subjects could take as long as needed to respond, as we did not put an emphasis on speed. The orientation of both target and flankers, as well as the display hemifield, was randomly selected in each trial. There were 40 trials for each combination of cue condition (neutral vs. valid), target–flanker spacing (1.1°, 2°, 3°, 4°, 5°, 6°, 8°, and uncrowded), and flanker arrangement (tangential vs. radial). Trial order was unpredictable (quasi-randomized). In total, the experiment consisted of 1280 trials, which were divided into two sessions of 640 trials each. Participants rested for half an hour between the two sessions. Each session was further divided into 10 blocks. Following each response, a high- or low-pitched tone played to indicate a correct or incorrect response, respectively. Note that participants completed 40 practice trials prior to beginning the actual experiment.

### Analysis

Data collected from both the right and left hemifields were pooled together. A three-way repeated-measures analysis of variance (ANOVA), with cue condition × target–flanker spacing × stimuli arrangement (radial vs. tangential), was performed on the accuracy data, excluding the trials where the target appeared without flankers (uncrowded). Additionally, and as a secondary dependent variable, we performed this same analysis on reaction time (RT) in correct response trials to rule out speed–accuracy tradeoffs. Next, individual accuracy data were fitted to a Weibull function (Weibull, 1951) with the goal of computing critical spacing thresholds for each condition (valid cue and neutral cue). Following previous studies (Rosen, Chakravarthi, & Pelli, 2014), we summarized the Weibull function by calculating the threshold spacing (i.e., 75% of correct trials in two-alternative forced choice), which provided us with our critical spacing values. Finally, using the critical spacing data, we conducted a 2 × 2 repeated-measures ANOVA to explore the relationships among cue condition, display arrangement, and critical spacing. Follow-up repeated-measures *t*-tests were performed to further parse out condition differences in critical spacing. The data and analysis codes are available from the corresponding author upon request.

## Results

We excluded from analysis all trials in which participants broke fixation during the stimulus presentation (<0.5% of all trials).

### Accuracy

**Figure 2.**
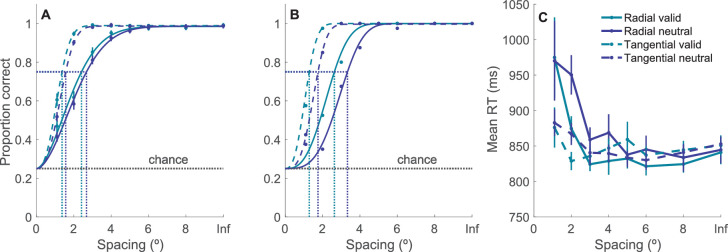
Performance as a function of critical spacing. (A) Mean proportion correct and fitted Weibull function. (B) Proportion correct and fitted Weibull function of one participant. This model was used to estimate the critical spacing of each condition. Dotted vertical lines indicate the critical spacing for both valid cue and neutral cue conditions and radial and tangential layouts. (C) Mean RTs as a function of target–flanker spacing. Inf, infinite spacing represents uncrowded display trials. Error bars: ±1 within-subject standard error (Morey, 2008).

Figures 2A and 2B plot the mean accuracy rate (Figure 2A) as a function of target–flanker spacing for two cue types (neutral and valid) and two flanker arrangements (radial and tangential). As expected, we found a significant main effect for cue condition, *F*(1, 15) = 25.31, *p* < 0.001, η^2^*_p_* = 0.63, showing that participant accuracy was higher during valid cue trials than neutral cue trials, with 0.87 ± 0.02 (*M* ± *SE*) and 0.83 ± 0.02, respectively. We also found a significant main effect for the target–flanker spacing, *F*(6, 90) = 266.88, *p* < 0.001, η^2^*_p_* = 0.95, which, in agreement with previous research, showed that accuracy increased as target–flanker spacing increased. Additionally, there was a significant main effect for stimuli layout (radial vs. tangential), *F*(1, 15) = 104.84, *p* < 0.001, η^2^*_p_* = 0.87, showing that accuracy was higher in tangential display trials than in radial display trials, with 0.9 ± 0.01 and 0.8 ± 0.02, respectively. Next, a significant interaction was found between cue condition and target–flanker spacing, *F*(6, 90) = 3.38, *p* < 0.005, η^2^*_p_* = 0.18, which revealed that the impact of spacing on accuracy varied across cue conditions. Another significant interaction effect was found between stimuli layout (radial vs. tangential) and target–flanker spacing, *F*(6, 90) = 32.23, *p* < 0.001, η^2^*_p_* = 0.68, which revealed that the impact of spacing on accuracy varied across stimuli layout. Interestingly, the interaction between cue condition and stimuli layout was not significant (*p* = 0.57). Furthermore, the three-way interaction among target–flanker spacing, cue condition, and stimuli layout was not significant (*p* = 0.3). We further explored these results by fitting the data to an exponential curve and calculating the critical spacing for each condition.

### Reaction times

Figure 2C plots mean RTs as a function of target–flanker spacing for two cue types (neutral and valid) and two flanker arrangements (radial and tangential). We found a significant main effect for the target–flanker spacing, *F*(6, 90) = 4.28, *p* < 0.001, η^2^*_p_* = 0.22, which, in agreement with the accuracy data, showed that performance (i.e., RT speed) increased as target–flanker spacing increased. Additionally, there was a significant interaction between stimuli layout (radial vs. tangential) and cue condition (valid vs. neutral), *F*(1, 15) = 5.16, *p* < 0.05, η^2^*_p_* = 0.26, and between stimuli layout and target–flankers spacing, *F*(1, 15) = 3.36, *p* < 0.01, η^2^*_p_* = 0.18. No other effect was significant with RTs (all *p* > 0.05). Importantly, the analysis of RT data confirmed that the results on accuracy data were not due to speed–accuracy tradeoffs.

### Critical spacing

**Figure 3.**
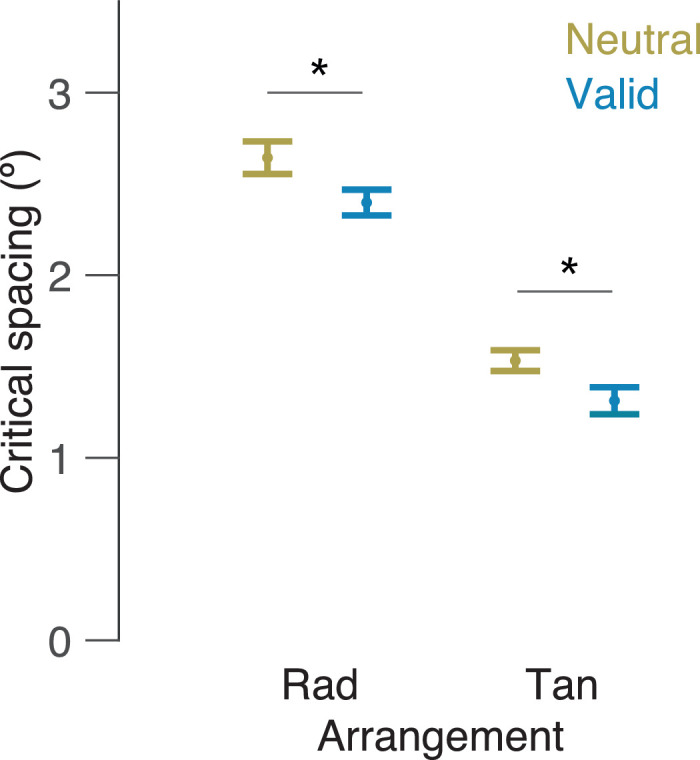
Crowding window. Mean critical spacing values in degrees as a function of cue condition and flanker arrangement. Error bars: ±1 within-subject standard error (Morey, 2008).

Two participants had to be removed from further analysis because their data did not reach asymptote (i.e., the estimated critical spacing was exceptionally large). Figure 3 plots the critical spacing for the radial and tangential arrangements in the form of their horizontal and vertical extent of crowding (crowding window) for neutral and valid trials. As expected, there was a main effect of cue condition on critical spacing, *F*(1, 13) = 18.38, *p* = 0.001, η^2^*_p_* = 0.58, where we saw smaller critical spacing (18% less) in valid trials than in neutral trials. As expected, there was a main effect of display arrangement on the critical spacing, *F*(1, 13) = 121.35, *p* = 0.0001, η^2^*_p_* = 0.90, where we found smaller critical spacing in the tangential rather than in the radial arrangements. Importantly, there was no interaction between cue condition and display arrangement (*p* = 0.81).

## Discussion

The present study examined the combined effects of transient attention and flanker arrangement on the crowding window. Specifically, we measured the effect of a cue on the critical spacing for both tangential and radial flanker arrangements. The results showed that both a peripheral cue and a tangential arrangement reduced the critical spacing. Importantly, our findings also showed that attention affected the critical spacing for both arrangements to the same extent.

### Locus of attention and crowding asymmetries

Previous studies have shown that spatial attention, or processes that are affected by spatial attention (e.g., visual resolution), play an important role in the inner–outer asymmetry (Kewan & Yashar, 2021; Petrov & Meleshkevich, 2011a). In contrast, in the present study we showed that directing spatial attention produced effects of similar magnitude on the radial and tangential arrangement, indicating that spatial attention may not modulate radial–tangential anisotropy. Thus, our findings suggest that radial–tangential anisotropy is due to processes that are unrelated to transient attention. Furthermore, the results of the present study, together with those of Kewan and Yashar (2021), suggest that different processes may be involved in inner–outer asymmetry versus radial–tangential anisotropy.

### Models of attention and crowding

Our findings are consistent with recent models of attention. Spatial attention enhances various aspects of stimulus representation, such as contrast, signal-to-noise ratio, and visual acuity (Dosher & Lu, 2000; Herrmann, Montaser-Kouhsari, Carrasco, & Heeger, 2010; Ling & Carrasco, 2006; Montagna, Pestilli, & Carrasco, 2009; Pestilli & Carrasco, 2005). This signal enhancement can explain the overall increase in correct valid cue trials but not the reduction in critical spacing. Therefore, a possible explanation for our finding could be that attention increases visual spatial resolution in the periphery (Yeshurun & Carrasco, 1998). Neurophysiological studies provide support for this interpretation, as they show that attention leads to contraction of the receptive field of cells around the attended location (e.g., Desimone & Duncan, 1995; Luck, Chelazzi, Hillyard, & Desimone, 1997; Moran & Desimone, 1985; reviewed by Anton-Erxleben and Carrasco (2013). This attentional effect reduces the receptive field size over the target area, which may also reduce the pooling area or “integration field” of crowding (Pelli et al., 2004). In this way, target and flankers would no longer fall within the same integration field. Our results show that this reduction is uniform across the radial and tangential axes.

Our findings may also be consistent with the attentional selection view. According to this view, attentional resolution is much coarser than visual spatial resolution, leading to the joint selection of target and flankers (Intriligator & Cavanagh, 2001; Tripathy & Cavanagh, 2002). This theory is compatible with our findings only if we assume that attentional selection and pre-cueing transient attention involve separate processes. The former is a top–down (endogenous) process, whereas the latter is bottom–up (exogenous) driven. Indeed, recent studies suggest that endogenous and exogenous attention vary in how they affect contrast sensitivity and visual resolution (Fernández, Okun, & Carrasco, 2021; Jigo, Heeger, & Carrasco, 2021). This evidence indicates that the pre-cue effect reflects processes that are less constrained and therefore different from target selection, which is limited in nature. Thus, although transient attention increased spatial resolution, the top–down selection process was still limited by its lower resolution in the visual periphery, resulting in only a small reduction in the critical spacing at the cue location. Some studies suggest that there may not be a capacity limit to the effects of peripheral pre-cueing on visual sensitivity (Solomon, 2004; Solomon & Morgan, 2018). However, further work is required to determine whether the pre-cueing effect on the critical spacing is capacity limited or not, such as by simultaneously pre-cueing multiple locations in a crowded display.

### Limitations

Our study did not directly explore the differences between the involvement of attention in radial–tangential anisotropy and its noninvolvement in inner–outer asymmetry. Accordingly, further investigation is required to more concretely disassociate these two phenomena. First, the distance between the inner and outer flankers and the target was kept equal throughout the task, which did not take into account the inner–outer asymmetry found in crowding (Petrov et al., 2007). Given that the outer flanker tends to increase crowding more than the inner flanker, future studies should explore how attention affects crowding when the distance between the inner flanker and the target and between the outer flanker and the target differ. Additionally, we kept the eccentricity of the stimuli constant. However, in a tangential arrangement, the magnitude of the cueing effect varies with target eccentricity (Yeshurun & Rashal, 2010). Thus, future studies should explore how peripheral cues influence crowding at different eccentricities, for both radial and tangential arrangements.

## Conclusions

The present study shows that the effect of spatial attention on the spatial extant of crowding is isotropy—namely, attention reduced the critical spacing for both radial and tangential arrangements to the same extent. Our results extend previous attentional findings on tangential and radial target–flanker arrangements and suggest that the locus of attention plays the same role in both. Furthermore, we provide evidence in support of the view that attention enhances spatial resolution by contracting receptive (or integration) field size, and we suggest that this contraction is uniform across the radial and tangential axes.
